# Inflammatory Mediators and Angiogenic Factors in Choroidal Neovascularization: Pathogenetic Interactions and Therapeutic Implications

**DOI:** 10.1155/2010/546826

**Published:** 2010-08-25

**Authors:** Claudio Campa, Ciro Costagliola, Carlo Incorvaia, Carl Sheridan, Francesco Semeraro, Katia De Nadai, Adolfo Sebastiani, Francesco Parmeggiani

**Affiliations:** ^1^Department of Ophthalmology, University of Ferrara, Corso Giovecca 203, 44121 Ferrara, Italy; ^2^St Paul's Eye Unit, Royal Liverpool University Hospital, Liverpool, UK; ^3^Department of Health Sciences, University of Molise, Campobasso, Italy; ^4^Department of Ophthalmology, University of Brescia, Brescia, Italy; ^5^Center for Retinitis Pigmentosa of Veneto Region, ULSS 15 Alta Padovana, Camposampiero Hospital, Camposampiero, Italy

## Abstract

Choroidal neovascularization (CNV) is a common and severe complication in heterogeneous diseases affecting the posterior segment of the eye, the most frequent being represented by age-related macular degeneration. Although the term may suggest just a vascular pathological condition, CNV is more properly definable as an aberrant tissue invasion of endothelial and inflammatory cells, in which both angiogenesis and inflammation are involved. Experimental and clinical evidences show that vascular endothelial growth factor is a key signal in promoting angiogenesis. However, many other molecules, distinctive of the inflammatory response, act as neovascular activators in CNV. These include fibroblast growth factor, transforming growth factor, tumor necrosis factor, interleukins, and complement. This paper reviews the role of inflammatory mediators and angiogenic factors in the development of CNV, proposing pathogenetic assumptions of mutual interaction. As an extension of this concept, new therapeutic approaches geared to have an effect on both the vascular and the extravascular components of CNV are discussed.

## 1. Introduction

Choroidal neovascularization (CNV) represents the growth of new blood vessels from the choroid into the subretinal pigment epithelium which, in several patients, reaches the retina. CNV is a common pathological endpoint in a heterogeneous variety of chorioretinal diseases [1]. Virtually any pathologic process that involves the retinal pigment epithelium (RPE) and damages Bruch's membrane can be complicated by CNV. The most frequent cause of CNV is age-related macular degeneration (AMD) [2].

**Figure 1:**
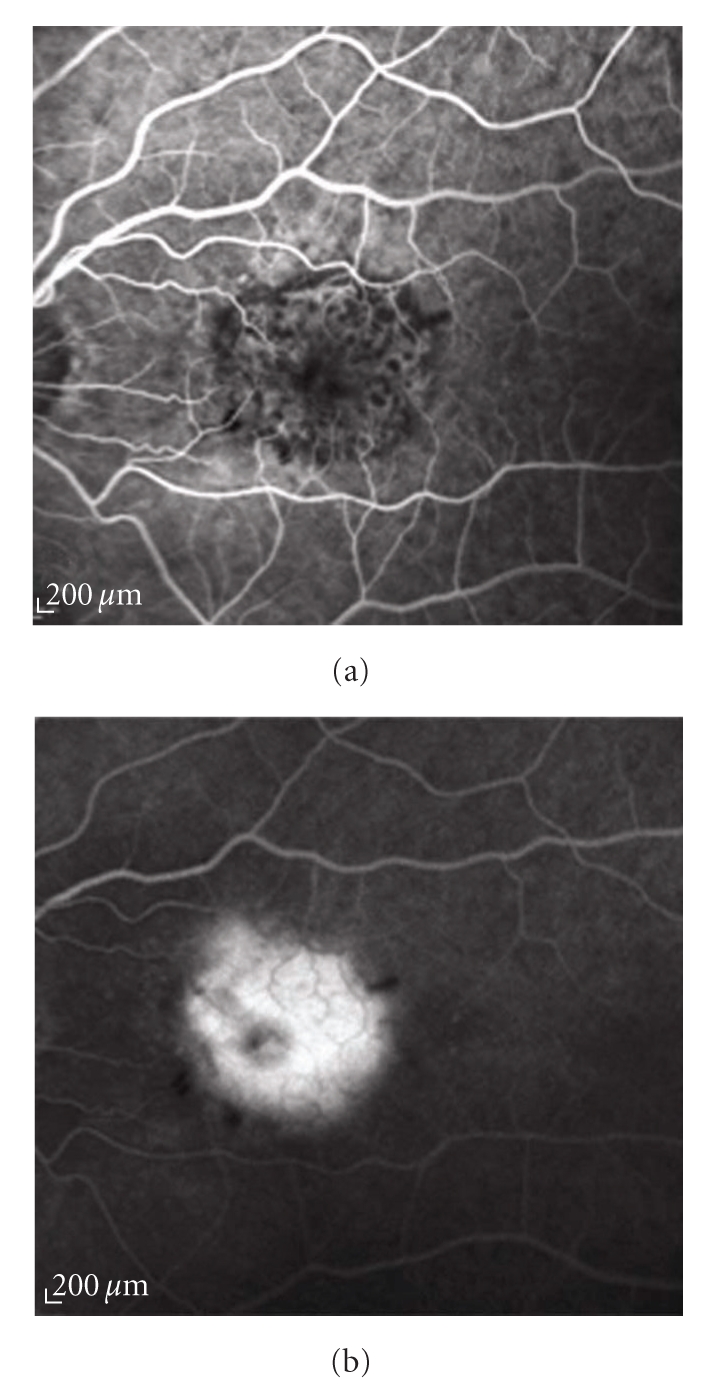
Fluorescein angiography of a classic choroidal neovascularization. (a) Early and (b) late angiograms: the lesion is characterized by a well demarcated area of early fluorescence with a progressive leakage of the dye to the subretinal space leading to blurring of the borders in the late phase of the exam.

**Figure 2:**
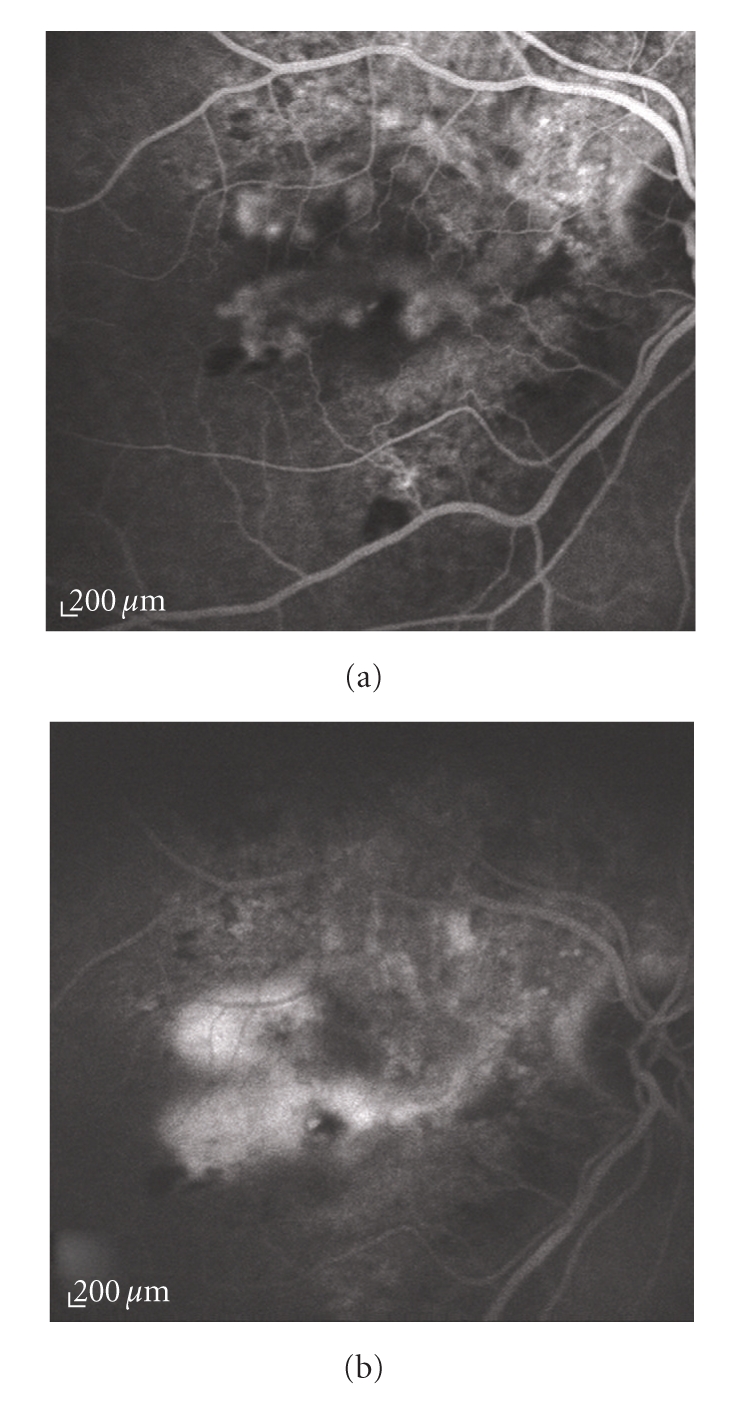
Fluorescein angiography of a predominantly classic choroidal neovascularization. (a) Early and (b) late angiograms: the lesion has a mixture of angiographic features of the classic and occult type, with the classic component making up more than 50% of the entire neovascular complex.

**Figure 3:**
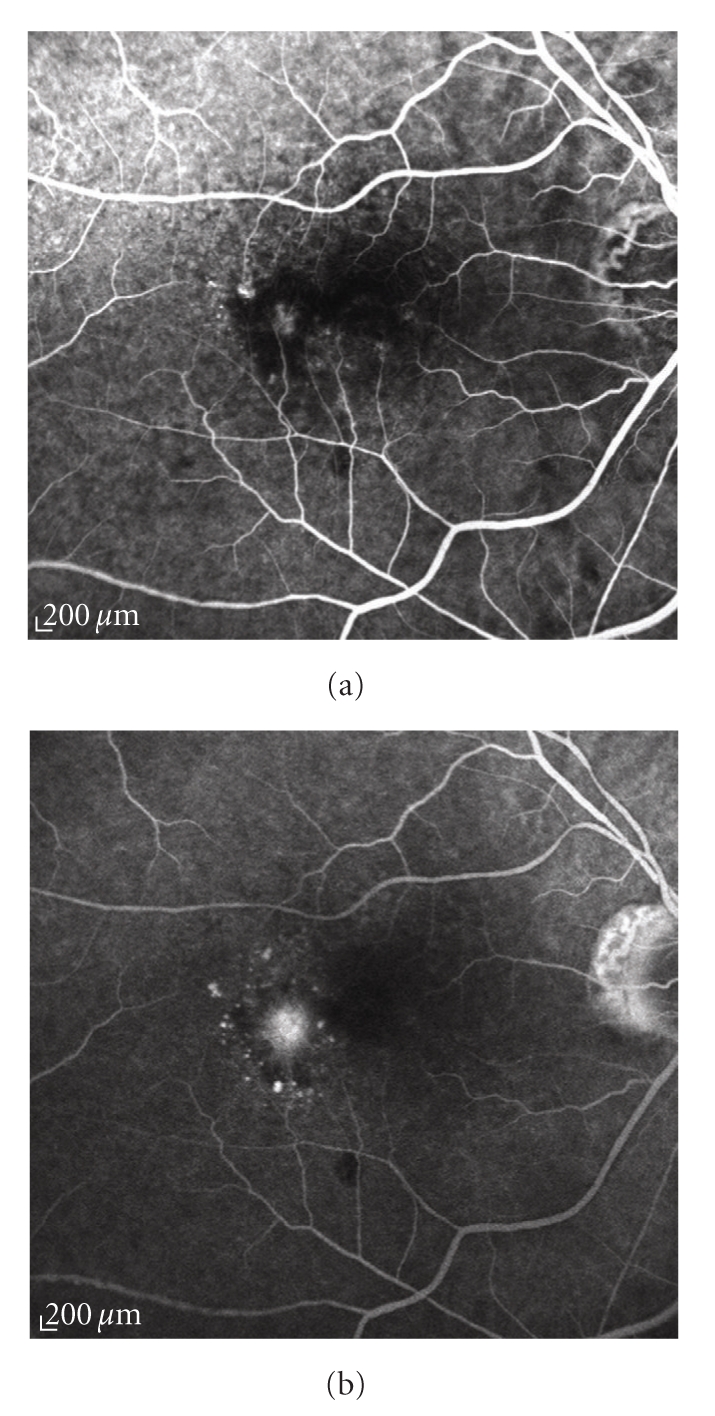
Fluorescein angiography of a minimally classic choroidal neovascularization. (a) Early and (b) late angiograms: the lesion has a mixture of the angiographic features of the classic and occult type, with the classic component making up less than 50% of the entire neovascular complex.

**Figure 4:**
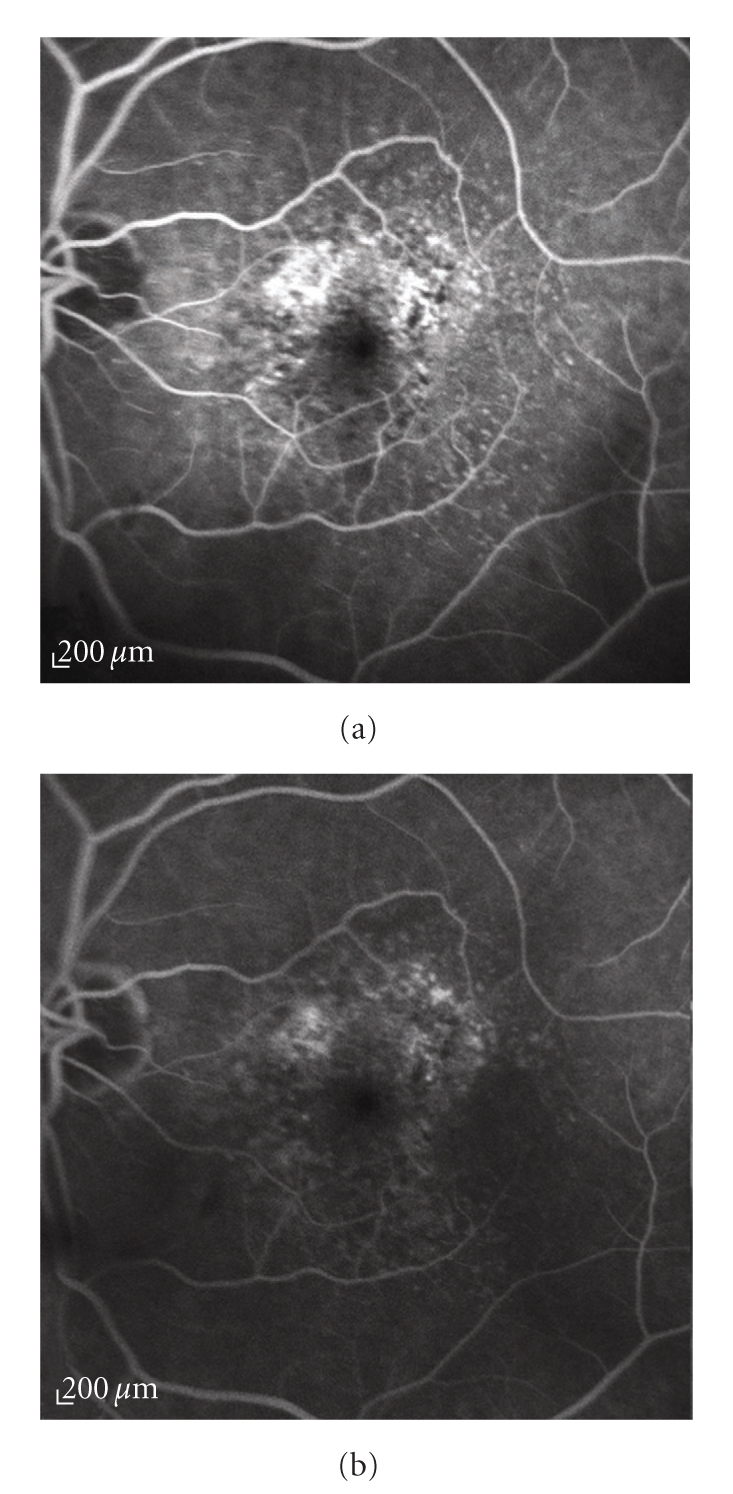
Fluorescein angiography of an occult choroidal neovascularization. (a) Early and (b) late angiograms: the lesion appears within 1-2 minutes from the start of the exams and persists during the late phase; it is characterized by areas of irregular elevation of the retinal pigment epithelium that present stippled hyperfluorescence.

The clinical classification of AMD-related CNV is carried out according to the definitions of Treatment of Age-Related Macular Degeneration with Photodynamic Therapy (TAP) and Visudyne in Photodynamic Therapy (VIP) studies [3–6], distinguishing between four subtypes characterized by different patterns during the fluorescein angiography (FA):

- *classic CNV:* a demarcated area of uniform hyperfluorescence with a hypofluorescent margin in FA early phase, and dye leakage obscuring the boundaries during the mid and late phases (Figures 1(a) and 1(b));
- *predominantly classic CNV*: the classic component occupying 50% or more of the entire neovascular lesion (including occult CNV and all the fluorescence-blocking constituents) (Figures 2(a) and 2(b));
- *minimally classic CNV*: the classic component occupying less than 50% of the neovascular complex (Figures 3(a) and 3(b));
- *occult CNV with no classic component*: including two types: (*a*) fibrovascular RPE detachment appearing as stippled hyperfluorescence with irregular RPE elevation (Figures 4(a) and 4(b)); (*b*) an undefined area with a late-phase dye leakage from an undetermined source, not corresponding to classic CNV and/or fibrovascular RPE detachment in FA early or mid phases.

Correspondingly, three basic patterns of CNV growth have been described [7]:

- sub-RPE (type 1, clinically definable as occult CNV with no classic component);
- subretinal (type 2, clinically definable as classic CNV);
- sub-RPE and subretinal (combined, clinically definable as predominantly classic or minimally classic CNV).

## 2. Pathogenesis of Choroidal Neovascularization

In the surgically excised CNV of patients with AMD, the histopathologic examination indicates the presence of fragments of Bruch's membrane, RPE, photoreceptors, vascular endothelium, fibroblasts, macrophages, circulating progenitor/stem cells, and extracellular components including collagen, fibrin, and basal laminar deposits [8–14]. Hence, a two-component model for CNV has been proposed to describe CNV: (*i*) the vascular component of CNV is composed of vascular endothelial cells (ECs), pericytes and precursors of EC; (*i* *i*) the extravascular component is comprised of inflammatory cells (macrophages, lymphocytes, granulocytes, and foreign body giant cells), glial cells, RPE cells and fibroblasts [15]. Therefore, CNV is a process with a pathogenesis involving both inflammation and angiogenesis. The relevance of each component depends on both the underlying disease and dynamic stage of CNV development.

The natural history of CNV may be schematically divided in three stages: in the initiation stage, EC are derived from the chriocapillaris proliferate and migrate towards the retina through the Bruch's membrane; subsequently, during the active stage, the neovascular complex grows up to a certain size; finally, for reasons not yet well-known, the CNV becomes fibrotic and forms a disciform scar in the involutional stage [7].

### 2.1. Initiation Stage

#### 2.1.1. Vascular Endothelial Growth Factor

In the initiation stage of the neovascular lesion, it is now well established that vascular endothelial growth factor (VEGF) plays a key role as inciting stimulus involved in CNV development. VEGF production has a number of potential sources, counting EC, pericytes, glial cells, Müller cells, ganglion cells, photoreceptors, and RPE [16–20]. Particularly, RPE secretes VEGF in a polarized manner, with higher basal secretion towards Bruch's membrane than apical secretion towards in situ photoreceptors [21]. VEGF-A, commonly named simply VEGF, is the prototype member of a gene family, also including placenta growth factor (PlGF), VEGF-B, VEGF-C, VEGF-D, and orf-virus-encoded VEGF-E [22, 23]. The increased VEGF production is mainly determined by hypoxic stimuli, and VEGF per se triggers the growth of vascular EC derived from arteries, veins, and lymphatics. VEGF is also a survival factor for endothelium; it enhances microvascular permeability and promotes monocyte chemotaxis [24].

Alternative exon splicing results in the generation of four main VEGF isoforms, named VEGF-121, VEGF-165, VEGF-189, and VEGF-206 basing on the number of amino acids after the signal sequence of slicing [24]. The smaller isoforms (VEGF-121 and VEGF-165) are freely diffusible whereas the larger ones (VEGF-189 and VEGF-206) are bound to heparin-containing proteoglycans on the cell surface or basement membrane [25]. These longer molecules can be released, after a plasmin-related proteolytic cleavage, as diffusible bioactive fragments [25, 26]. VEGF-165 can be proteolyzed by various matrix metalloproteinases (MMPs), especially the MMP-3, generating VEGF-113 which seems to be very similar to the plasmin-generated VEGF fragments [27].

Hitherto, three VEGF-receptors (VEGFRs) have been identified: (*i*) VEGFR-1 was firstly identified more than fifteen years ago, but its function is still debated [24, 28]; (*i* *i*) VEGFR-2 is considered the major mediator of the mitogenic, angiogenic and propermeability effects of VEGF [24]; (*i* *i* *i*) VEGFR-3 is identical to neuropilin-1, implicated in axon guidance as a receptor for collapsin/semaphorin molecules, and it appears to enhance the effectiveness of the signal transduction mediated by VEGFR-2 [24].

Experimental and clinical evidences indicate the central role of VEGF in CNV occurrence. In fact, VEGF is present in surgically excised CNV [16, 17, 29–31], and vitreous levels of VEGF were significantly higher in patients with neovascular AMD compared with healthy controls [32, 33]. The VEGF overexpression in mice's RPE is sufficient to cause the development of CNV [34, 35], and, in animal models, VEGF blocking treatments are able to cure the laser-induced CNV [36, 37]. Finally, intravitreal injections of VEGF elicit proliferation of choroidal EC in nonhuman primates [38]. In physiologic and pathophysiologic conditions, the upregulation of proangiogenic cytokine expression, such as VEGF, is involved in the response to tissue hypoxia that it is mediated by the family of transcription regulators, named hypoxia inducible factors (HIFs). It is therefore plausible that hypoxia represents a major stimulus for the submacular wound healing, and, within this context, CNV is other than one element of this process which, in turn, leads to HIF synthesis; HIF are the key signals for hyperproduction of VEGF with subsequent aberrant growth of the neovascular component of submacular tissue repair. In fact, the presence of HIF-1alpha and HIF-2alpha has been recently discovered in active CNV specimens [39]. In the ocular tissues, VEGF acts as both a specific EC mitogen and promoter of vascular permeability. However, in addition to angiogenesis, vasculogenesis appears to be implicated in CNV development, indeed: (i) VEGF is a chemoattractant for EC precursors, inducing their mobilization and promoting their differentiation [40]; (ii) chemokine stromal cell-derived factor 1-alpha and its receptor CXCR4, both involved in the recruitment of EC precursors, have been detected inside CNV [41]. VEGF induces CNV enlargement also by stimulating EC expression of MMPs, which degrade the extracellular matrix and facilitate neovascular tissue invasion [42]. Lastly, VEGF represents a potent chemotactic signal for macrophages [43, 44].

#### 2.1.2. Macrophages and Other Cytokines

Macrophages are other important players in the process of CNV development. In experimental model of CNV, macrophages depletion diminishes both size and severity of the neovascular lesion [45]. Leukocytes are recruited in CNV not only by VEGF but also by vascular adhesion protein-1, an EC adhesion molecule [46]. It is unknown whether either macrophages actively cause breaks in Bruch's membrane (via production of collagenase/elastase), or they are introduced into the ill area after the CNV breaks of Bruch's membrane [8]. The arriving of macrophages elicits the production of tumor necrosis factor-*α* (TNF-*α*) which, in particular, stimulates the synthesis of type-8 interleukin (IL), monocyte colonization protein, and RPE-secreted VEGF [11]. This autocrine/paracrine loop is completed with the recruitment of further macrophages by type-1 monocyte colonization protein [47]. At the level of RPE cells, integrins *α*3 and *α*5 are expressed, mediating the migration of vascular ECs or macrophages in the early stage of CNV development [48]. As well, IL-2, IL-6, and IL-10 might participate to CNV expansion, but their exact roles have not been deeply investigated yet [49–51].

#### 2.1.3. Angiogenic and Antiangiogenic Agents

Several angiogenic/antiangiogenic molecules, different from VEGFs, are implicated in CNV development [52].

- Insulin-like growth factor is a mediator of anabolic and mitogenic actions of the growth hormone; inside AMD-related CNV present are mRNAs of this factor and of its receptor [53]; in vivo, both VEGF expression and CNV activity are downregulated by a specific receptorial inhibitor (picropodophyllin) [54].
- Nitric oxide is a signaling molecule with pleiotropic effects, and it is a well-known mediator of vascular dilatation and permeability [55]; experimental findings indicate that nitric oxide is an important CNV stimulator, and that its reduction, obtained in pharmacologic or genetic manner, represents a potential therapeutic strategy for CNV [56].
- Angiostatin is a plasminogen fragment firstly recognized as endogenous angiogenic inhibitor [57]; subretinal injection of recombinant adenoassociated virus vector expressing mouse angiostatin is able to suppress experimental CNV in a rat model [58].
- Endostatin, another endogenous inhibitor of angiogenesis, is generated by the cleavage of a collagen XVIII fragment [59]; in mice, intravenous administration of adenoviral vectors containing an expression construct for endostatin results in prevention of laser-induced CNV [60].
- Pigment epithelium-derived factor (PEDF) is a potent antiangiogenic and neuroprotective protein, normally produced by RPE [61]; in pigs and mice, the periocular injection of an adenoviral vector encoding PEDF inhibits CNV development [62, 63].
- CCR3 (also known as CD193) is a chemokine receptor best known for its role in promoting eosinophil and mast cell trafficking; it is specifically expressed in CNV endothelial cells in humans with AMD [64]. CCR3 targeting reduces CNV in a mouse model through a direct antivascular effect which does not appear to involve modulation of cellular inflammation [64].

### 2.2. Active Stage

In the course of the natural history of CNV, active stage is characterized by the progressive enlargement of neovascular complex. This neovascular enlargement is mainly related to the presence of many inflammatory cells, synergistically acting with aberrant cytokines produced via autocrine/paracrine mechanisms. Vascular endothelium and macrophages produce MMPs which, in turn, degrade extracellular matrix allowing CNV infiltration through Bruch's membrane [8]. Several MMPs were detected in the vitreous of AMD patients and in surgically excised subfoveal CNV [65, 66]. Particularly, peculiar MMPs seem to play a key role in CNV growth; in fact: (*i*) MMP-2/MMP-9 deficient mice have more difficulty in developing laser-induced CNV [67, 68]; (*i* *i*) overexpression of tissue inhibitor for MMP-3 (TIMP-3) in RPE cells reduces the activity of experimental CNV [69]. At the same time, macrophages express tissue factor, a protein involved in fibrinogenesis, which is able to generate a fibrin scaffold for the growing of CNV [8]. During active stage, also angiopoietins and their receptors (Tie-1, Tie-2) play a role in angiogenic process. Histological examination of AMD-related CNV showed that angiopoietin-1 and -2 and Tie-2 were present [70]. Angiopoietins are essential for maturity and integrity of vessels: angiopoietin-1 appears crucial for this structural stabilization in normal vascular system whereas the dramatic upregulation of angiopoietin-2, in presence of VEGF, is a prerequisite for vascular remodeling and/or normal angiogenesis [71]. Moreover, at this stage, RPE produces a basic fibroblast growth factor (FGF-2), a heparin-binding peptide, that stimulates pathologic angiogenesis. However, FGF-2 expression alone is neither necessary nor sufficient for CNV development [71, 72]. Transforming growth factor-beta (TGF-*β*) is another important factor secreted by RPE during active CNV phase [73]; since it is a potent inducer of extracellular matrix synthesis, reliably it limits the extent of neovascular complex, starting that process then resulting in progressive CNV fibrosis [8]. Recent experimental evidences suggest a role also for hepatocyte growth factor (HGF), a mitogen for hepatic and renal cells that may be involved in CNV progression [74]; whether this is a result of its dedifferentiating effect on RPE cells is currently unclear.

Activation of complement, specifically the formation of membrane attack complex (MAC), is essential for the development of laser-induced CNV in mice [75] underlining, once again, the importance of inflammation in the pathogenesis of the neovascular complex. MAC can mediate the release of several factors from various nucleated cells, such as VEGF, FGF-2, and platelet-derived growth factor (PDGF), with consequent amplification of the angiogenic processes [75]. Gene variants of the complement components have been evaluated as risk factors for AMD among different ethnic clusters [76–101]. Hitherto, one of the most investigated single nucleotide polymorphism (SNP) has been the rs1061170, which causes a Tyr402His amino acid substitution in complement factor H (CFH). Independent studies have documented that the allele 402His is related to increased incidence and/or severity of AMD in several populations [76–100, 102]. The frequency of 402His allele varies greatly between populations and, notionally, it may contribute to the observed variability in the incidence of AMD among different ethnic groups [103]. Finally, also elevated plasma levels of C3a complement compound in patients with AMD-related CNV have recently suggested the presence of an association between systemic complement activation and this pathologic occurrence [104].

In the course of initiation stage, the neovascularization is highly dependent on VEGF but, afterward, it undergoes a process of maturation which makes it less dependent on this growth factor. In fact, once the new vessels are formed, ECs start to secrete other factors to recruit mural cells (pericytes) that promote vessel stabilization, endothelium differentiation, and growth arrest. The most important of these factors is PDGF-B which works through its receptors expressed by pericytes [105–107]. In animal models of CNV, inhibition of both VEGF-A and PDGF-B signaling appeared more effective for vessel regression than blocking VEGF-A alone [108].

### 2.3. Involutional Stage

Whilst there is considerable overlapping between the phases of CNV progress, at certain time the balance of these pathologic events shifts towards antiangiogenic and antiproteolytic activities, resulting in the involutional stage of CNV [8]. Indeed, it is quite frequent to find different developmental stages within the same neovascular complex, mostly characterized as either cellular or fibrotic regions [14]. At this stage, the most important players are TGF-*β* and TIMP-3, produced by RPE, which are able to markedly influence both the secretion of extracellular matrix and the tissue remodeling. Concurrently, angiogenesis continues until a state of normoxia or hyperoxia exists, thereby switching off VEGF synthesis. The outcomes of these processes are the maturation of established vessels and the occurrence of scar tissue. The origin of vascular elements contributing to the subretinal fibrosis is not yet clear, but it is known that RPE cells themselves, directed by TNF-*α*, TGF-*β*, and other growth factors, dedifferentiate and proliferate showing, together with choroidal fibroblasts, a wound repair pattern [109].

## 3. Therapeutic Approaches for Choroidal Neovascularization

The wider knowledge of the mechanisms involved in the pathogenesis and development of CNV has led, in the last few years, to a remarkable increase of the possible pharmacological strategies toward this severe macular lesion (Table 1).

### 3.1. Therapies Directed against the Vascular Component of Choroidal Neovascularization

In view of the fact that VEGF plays a key role in the pathogenesis of CNV, targeting VEGF has soon appeared as an effective strategy to treat CNV [110]. Antivascular endothelial growth factor (anti-VEGF) drugs not only can arrest choroidal angiogenesis, but they also reduce vascular hyperpermeability which, in patients with CNV, is often the main cause of visual acuity (VA) deterioration.

Pegaptanib sodium and ranibizumab were the first intraocular anti-VEGF treatments evaluated in large, randomized, controlled clinical trials for the treatment of neovascular AMD. Both have been administered locally by repeated intravitreal injections.

Pegaptanib sodium is a pegylated oligonucleotide aptamer that selectively binds to and inactivates just the VEGF-165, the most abundant isoform of VEGF. In the VISION trial—a phase III double-masked, sham-controlled, dose-ranging study including all lesion subtypes and dimensional categories—pegaptanib sodium prevented moderate vision loss (the primary endpoint, which was defined as loss of <15 letters of vision) in 70% of the treated patients compared with 55% for the control group at one year. On average, patients in the pegaptanib sodium group lost 8 letters at one year, compared with a loss of 15 letters in the sham injection group. The proportion of patients who experienced a moderate gain in vision (defined as a change of ≥15 letters at one year from baseline) was small in both groups −6% in the pegaptanib sodium group versus 2% in the sham-injection group [111]. In two years, 59% of eyes treated with pegaptanib lost less than 15 letters compared with 45% of sham-treated eyes [112].

 Ranibizumab is a recombinant, humanized antibody fragment that binds to and potently neutralizes the biological activities of all known human VEGF isoforms, as well as the proteolytic cleavage product VEGF-110. In a large randomized, multicentre, sham-controlled phase III study (MARINA trial) that included only patients with minimally classic or occult CNVs, at the 12-month visit, 95% of ranibizumab-treated eyes maintained stable vision (within 15 letters) compared with 62% of sham-treated eyes. An improvement of VA by ≥15 letters was found in 34% of eyes treated as compared with 5% of the sham-injection group. After 24 months, 90% of eyes in the ranibizumab group versus 53% in the control group demonstrated stable vision. A mean improvement of 7 letters was documented at 12 and 24 months of follow up in the ranibizumab arms. Improved vision by ≥15 letters occurred in 33% of eyes treated with ranibizumab, and 42% of patients in this group obtained a final VA of 20/40 or better [113]. The ANCHOR study was designed as a prospective, randomized phase III trial including patients with predominantly classic CNV secondary to AMD. Repeated injections of ranibizumab were compared with an arbitrary treatment with photodynamic therapy. In one year, 96% of ranibizumab-treated eyes lost less than 15 letters versus 64% of photodynamic-treated eyes. An improvement ≥15 letters was found in 40% of eyes injected with ranibizumab compared with 6% in the other treatment group. A vision gain of ≥30 letters was achieved in 12% of ranibizumab-treated patients. Mean VA demonstrated an improvement of 11 letters in one year and a final VA of 20/40 or better was found in 39% of ranibizumab-treated patients [114]. Consistent with results obtained at the 12-month check, at the 24th month of follow up the VA benefit due to ranibizumab was statistically significant and clinically meaningful [115].

Bevacizumab is a full-length recombinant, humanized antibody binding to all VEGF isoforms. The drug was originally developed to target pathologic angiogenesis in tumors and was approved by the FDA for the treatment of metastatic colorectal cancer. It is widely used as off-label drug for the treatment of various ocular neovascular diseases, including CNV, because numerous clinical studies have documented the ability of this compound to reduce angiogenesis and vascular hyperpermeability rapidly following its intravitreal repeated administrations [116–126]. Both favorable efficacy and safety profiles and lower costs are the major arguments to consider for an off-label use of this drug [116–131].

Several other molecules targeting VEGF and its signaling pathway are in various stages of clinical development for the treatment of CNV [132].

VEGF-Trap is a fusion protein that combines ligand-binding elements taken from the extracellular domains of VEGFR-1 and VEGFR- 2 fused to the Fc portion of IgG [132]. Unlike pegaptanib, ranibizumab, and bevacizumab, which all act through inhibition of VEGF-A, VEGF-Trap is designed to inhibit all members of the VEGF family: VEGF-A, -B, -C, -D, PlGF-1 and -2. The CLEAR-IT 2 study (phase II, randomized, controlled, clinical trial) has demonstrated that VEGF-Trap is able to significantly decrease retinal edema and CNV size in patients with neovascular AMD [133].

Another method of blocking the effects of VEGF is through inhibition of the downstream tyrosine kinase cascade activated by the VEGF binding to its receptors. Tyrosine kinase inhibitors, interacting with VEGF receptors currently in early stages of clinical development, include the two topical pazopanib formulations (TG100801 and TG101095), the oral formulation vatalanib (formerly PTK/ZK), and the intravitreal formulation AL39324 [132].

The levels of VEGF in the ocular tissues may be reduced also acting on its gene expression via small interfering RNA (siRNA). Therapeutic approach with siRNA works by downregulating the production of certain proteins as a result of degradation of specific mRNA. Bevasiranib (formerly Cand5) is an siRNA targeting mRNA of VEGF: it is currently tested in a phase III clinical trial (COBALT study) for the treatment of neovascular AMD. Since bevasiranib just blocks the VEGF production, the amount of VEGF already present in the eye at the time of administration precludes an immediate impact of the drug on CNV. For this reason, bevasiranib is evaluated as a combination therapy together with an agent that binds VEGF (ranibizumab) with the aim of reducing the dosage of the latter [134].

A major limitation of the anti-VEGF therapy is the difficulty to maintain the neovessels regression. This phenomenon is ascribed to the progressive maturation of CNV which then becomes less dependent on VEGF. As mentioned above, PDGF plays a key role in this process of maturation through the recruitment of pericytes [105, 106]. E10030 is an anti-PDGF aptamer that strips the pericytes from the neovascular tissue rendering it highly sensitive to an anti-VEGF attack. In a phase I clinical study evaluating E10030 in association with an anti-VEGF drug for the treatment of AMD-related CNV, 85% of patients exhibited neovascular regression [135]. This effect is further supported by the fact that, in ocular angiogenesis models, the pharmacologic inhibition of PDGF binding to its receptor (PDGFR-*β*) plus an anti-VEGF agent result in the regression of the neovascularization [136].

Beyond the inhibition of angiogenesis there is a new approach in the vascular targeting strategies: the so-called vascular disabling treatment. Vascular disabling agents (VDAs) target endothelial cells of the already established neovascular tissue leaving other blood vessels relatively unscathed. Zybrestat (combretastatin A4 phosphate, CA-4-P) is poised to become the first therapeutic product in this novel class of drug candidates. Zybrestat has a dual mode of action, targeting both VE-cadherin, a junction protein that is important for endothelial cell survival, and the associated beta-catenin/AKT signaling pathway [137]. In experimental models, CA-4-P was able to suppress the development of VEGF-induced retinal neovascularization and to promote CNV regression, showing potential for both prevention and treatment of ocular neovessels [138].

### 3.2. Therapies Directed against the Extravascular Component of Choroidal Neovascularization

The development of CNV is a complex process in which both angiogenesis and inflammation take part. As previously covered, VEGF apart, many cytokines and inflammatory cells are involved in CNV pathogenesis and, thus, they represent a potential target for therapy.

Anecortave acetate is a modified steroid derivative without glucocorticoid activity. It inhibits protease synthesis, which is necessary for cell migration through nonvascularized extracellular matrix in response to angiogenic stimulation. Juxtascleral depot of anecortave acetate at 6-month intervals was statistically superior to vehicle in a monotherapy trial at both 12 and 24 months for maintenance of vision and inhibition of CNV growth in patients with AMD; moreover, in a comparative trial, it was comparable to photodynamic therapy with verteporfin for maintaining vision over a 24-month period [139]. However, additional data about efficacy/safety profile have been requested for the clinical approval of this drug.

Integrins are cell adhesion receptors involved in the linkage to extracellular matrix and to adjacent cells. Integrins antagonists have effectively inhibited CNV progression in animal models, suggesting that these molecules may be beneficial in the treatment of CNV [140, 141]. Single intravitreal injections of JSM6427, a highly potent and specific small antagonist of *α*5*β*1 integrin, were well tolerated in patients with neovascular AMD and showed evidences of biological activity in phase I clinical study [142].

Whilst it has been demonstrated that MMPs are the key enzymes involved in the degradation of the extracellular matrix and have been shown to contribute to the growth of CNV, the strategies developed to inhibit CNV by overexpression of tissue inhibitors of MMP-3 have been somewhat disappointing [143]. The glycoprotein thrombospondin-1 suppresses angiogenesis by acting as both an activator of transforming growth factor-*β*, and a negative regulator of MMP-9 activation, and an activator of apoptotic pathways [52]. Recently a peptide derived from type 1 thrombospondin repeat-containing protein WISP-1 (wispostatin-1) has been shown to have inhibitory effect in vitro as well as in vivo in ocular neovascularization [144].

Several other drugs, already in use for several inflammatory disorders, are currently under investigation as potential treatments for CNV. They include cyclooxygenase inhibitors, sirolimus, and infliximab [145–149]; in particular, for these latter two, the preliminary data seem to be more interesting. Sirolimus, also known as rapamycin, acts on the protein kinase mammalian target of rapamycin, which regulates cell growth and metabolism. In addition to its anti-inflammatory, antifibrotic, and antiproliferative activities, sirolimus inhibits also angiogenesis by decreasing VEGF and transforming growth factor-*β*1 and by downregulating hypoxia-inducible factor-1*α* [132]. Systemic rapamycin is able to inhibit retinal and choroidal neovascularizations in mice [146]. A phase II clinical study is ongoing to assess the safety and efficacy of intravitreal ranibizumab plus subconjunctival sirolimus versus intravitreal ranibizumab plus placebo in patients with treatment-naive subfoveal CNV secondary to AMD [147]. Infliximab is a chimeric human IgG1 with a mouse Fv variable fragment of high TNF-*α* affinity and neutralizing capacity. In vivo, intravenous infliximab has been indicated in the treatment of rheumatologic, gastrointestinal, and dermatologic diseases, and recent studies have described its efficacy in the treatment of chronic ocular inflammation. Preclinical trials have demonstrated a reduction in CNV size in mice intravitreally treated with infliximab. However, there seems to be a dose-response relationship in which low doses of anti-TNF-*α* decrease angiogenesis while high doses increase it [148, 149].

## 4. Conclusions and Perspectives

The pathogenesis of CNV represents a highly complex process where not only angiogenesis but also inflammation plays an important role. Nowadays, the most frequent utilized treatment for the different typologies of subfoveal CNV is based on the pharmacological block of VEGF, which can be combined with the selective laser photothrombosis of the lesion (photodynamic therapy with verteporfin) [6]. However, neither therapy is ideal; in fact, verteporfin protocol is not usually associated with a functional improvement, and intravitreal drugs acting against VEGF are estimated to substantially improve vision in less than a third of patients, with one-sixth of treated subjects still progressing to legal blindness. Furthermore, in an elderly population, often already at risk for cerebrovascular accidents, there are concerns about possible systemic thromboembolic complications with repeated high dosages of anti-VEGF compounds [150]. Numerous intravitreal injections over many years may be also relatively contraindicated in some patients, such as diabetics, in who the underlying disease may favor infections and slow down the healing of the wound. In the last few years, following extensive immuno-histochemical and molecular biologic characterization of CNV, several innovative pharmacological treatments have come to notice. Although many of them are still in the early phase of development, it is likely that in the next future they will break new therapeutic ground in the treatment of CNV. Similar to cancer therapy, where a combination of agents have been found to be more effective than monotherapy, many retina specialists are starting to believe that a combination of two or more curative approaches will result in a better visual outcome than that of a single therapy for CNV. By targeting different mechanisms with individual agents, it should be possible to not only enhance efficacy, but also minimize unwanted collateral effects by using lower concentrations of each drug than those which would be used in monotherapy. In view of this new scenario, the relationships among pharmacogenetic predictors and diverse treatments towards CNV have been recently investigated considering the different responsiveness of subfoveal CNV to either photodynamic therapy with verteporfin [151–158] or intravitreal anti-VEGF agents [159, 160]. At present, it is very difficult to draw any unequivocal conclusion regarding the therapeutic influence towards CNV of those common immunologic gene polymorphisms, such as CFH Y402H and LOC387715/ARMS2 A69S, described as determinants of both phenotype and/or severity of AMD [76–101] and of the efficacy of very dissimilar CNV treatments [156–160], even if not in all published studies [161]. Unfortunately, there is no study in which has been verified the possible correlation between the allelic variants in complement cascade genes and the curative impact on CNV of immunotherapies, employed in single or combined modality, against the extravascular component of the lesion. Future investigations are warranted to outline these and other pharmacogenetic aspects about the possible treatments of subfoveal CNVs, stratifying the enrolled patients also on the basis of their different genotypic backgrounds for a remarkable optimization of each anti-CNV therapeutic strategy.

## Figures and Tables

**Table 1:** Therapeutic approaches for choroidal neovascularization.

Therapies directed against the vascular component of choroidal neovascularization.		
Agent	Class	Molecular target

Pegaptanib sodium	aptamer	VEGF-165

Ranibizumab	monoclonal antibody fragment	all VEGF isoforms

Bevacizumab	full-length monoclonal antibody	all VEGF isoforms

VEGF-trap	decoy receptor	all VEGF isoforms and PlGF

Pazopanib	tyrosine kinase inhibitors	VEGFR-1, VEGFR-2, VEGFR-3, PDGFR-*a*/*β*, and *c*-kit
TG100801		
TG101095		
Vatalanib		
AL39324		

Bevasiranib	siRNA	VEGF mRNA

E10030	aptamer	PDGF

Combretastat in A4 phosphate	vascular	VE-cadherin, beta-catenin/AKT

Therapies directed against the extra-vascular component of choroidal neovascularization		

Agent	Class	Molecular target

Anecortave acetate	corticosteroid	uPA, stromelysin (MMP-3)

JSM6427	integrins antagonist	*α*5*β*1 integrin

sirolimus	immunosuppressant	mTORC1

infliximab	Monoclonal antibody	TNF *α*

VEGF, vascular endothelial growth factor; VEGFR, vascular endothelial growth factor receptor; PlGF, placenta growth factor; PDGFR, platelet-derived growth factor receptor; PDGF, platelet-derived growth factor; uPA, urokinase plasminogen activator; MMP, matrix metalloproteinase; mTORC1, mammalian target of rapamycin complex 1.
